# Mendelian Randomization Analysis Support Causal Associations of HbA1c with Circulating Triglyceride, Total and Low-density Lipoprotein Cholesterol in a Chinese Population

**DOI:** 10.1038/s41598-019-41076-6

**Published:** 2019-04-02

**Authors:** Xu Jia, Yanan Hou, Min Xu, Zhiyun Zhao, Liping Xuan, Tiange Wang, Mian Li, Yu Xu, Jieli Lu, Yufang Bi, Weiqing Wang, Yuhong Chen

**Affiliations:** 1 State Key Laboratory of Medical Genomics, Key Laboratory for Endocrine and Metabolic Diseases of Ministry of Health, Shanghai National Clinical Research Center for Metabolic Diseases, and Collaborative Innovation Center of Systems Biomedicine, Rui-Jin Hospital, Shanghai Jiao-Tong University School of Medicine, Shanghai, 200025 China; 2 Shanghai Institute of Endocrine and Metabolic Diseases, Department of Endocrine and Metabolic Diseases, Rui-Jin Hospital, Shanghai Jiao Tong University School of Medicine, Shanghai, 200025 China

## Abstract

Previous observational studies supported a positive association of glycated hemoglobin A1c (HbA1c) level with serum triglyceride (TG), total cholesterol (TC), low-density lipoprotein cholesterol (LDL-C), and high-density lipoprotein cholesterol (HDL-C). However, the causal relationship between HbA1c and either one of them was unclear in the East Asians. We performed a Mendelian Randomization (MR) analysis in a community-based study sample in Shanghai, China (n = 11,935). To clarify the cause-and-effect relationships of HbA1c with the four interested lipids, an Expanded HbA1c genetic risk score (GRS) with 17 HbA1c-related common variants and a Conservative score by excluding 11 variants were built and adopted as the Instrumental Variables (IVs), respectively. The Expanded HbA1c-GRS was associated with 0.19 unit increment in log-TG (*P* = 0.009), 0.42 mmol/L TC (*P* = 0.01), and 0.33 mmol/L LDL-C (*P* = 0.01); while the Conservative HbA1c-GRS was associated with 0.22 unit in log-TG (*P* = 0.03), 0.60 mmol/L TC (*P* = 0.01), and 0.51 mmol/L LDL-C (*P* = 0.007). No causal relationship was detected for HDL-C. Sensitivity analysis supported the above findings. In conclusions, MR analysis supports a causal role of increased HbA1c level in increment of circulating TG, TC, and LDL-C in a Chinese population.

## Introduction

Glycated hemoglobin A1c (HbA1c) is a well-established clinical index for assessment of long-term glycemic regulation and diagnosis of diabetes^1^. Its predictive value for cardiovascular diseases (CVD) has been identified in both large-scale cohort study and Mendelian randomization (MR) analysis^2,3^. Meanwhile, compelling evidence showed that serum lipids, including triglyceride (TG), total cholesterol (TC), low-density lipoprotein cholesterol (LDL-C), and high-density lipoprotein (HDL-C), are associated with CVD risk due to their potential atherogenic effects. Among which, the elevated TG^4–6^ and LDL-C^7,8^ have been affirmatively proved as causal factors for coronary heart disease (CHD). On the other hand, HbA1c was extensively reported to be associated with the circulating lipid profile^9–11^. Lately, a trajectory analysis on youths with type 1 diabetes demonstrated that elevation of HbA1c from childhood into young adulthood may result in increases in LDL and non-HDL cholesterols^12^. In addition, results from large clinical trials identified that antidiabetic therapy can significantly reduce plasma TG, TC and LDL-C levels while effectively bringing down HbA1c level^13^. These findings may suggest causal relations of HbA1c with lipids; and bringing down HbA1c could become a therapeutic goal in treatment of dyslipidemia and CHD if such causalities been identified. Recently, a MR analysis performed in Europeans taking TG, LDL-C and HDL-C as interested outcomes proved a causal role of elevated HbA1c with a significantly increased HDL-C only^14^. While if such causality also exists in non-Caucasian populations has yet been identified; it draws forth an intriguing uncertainty as to the role of HbA1c in the changes of lipid profiles in East Asian populations.

Conventional observational studies are usually suffered by interference from confounders and reverse-causation^15^. Thus far, MR analysis has been broadly adopted in detecting causal effect of multiple risk factors for metabolic disorders^16–18^. In the MR analysis, genetic alleles were introduced to play the role of “representative” for the exposures, namely the Instrumental Variable (IV), to control such noises in causality detection. Since alleles are naturally randomized during gamete formation, and the genetic process from genotypes to phenotypes is definitely a one-way trip, the gene-outcome association is theoretically “immunized” from confounding effect or reversed causality^19^.

In the current study, we aimed to examine the causal relationships between HbA1c and serum lipids by performing MR analysis. The dataset was collected from community-based sample of 11,935 Chinese participants. To constrain the error rate in repeated statistical analysis, we composed the strength of multiple SNPs by setting up gene-exposure coefficient weighted genetic risk scores (GRS) as IVs^20,21^.

## Material and Methods

### Study Subjects

The current study was one part of the Risk Evaluation of Cancers in Chinese Diabetic Individuals: a Longitudinal (REACTION) Study, an ongoing nationwide prospective community-based cohort study involving 259,657 community dwellers, aged 40 years or older at baseline. The details of population selection were published in previous papers^17,22–24^.

The study participants of the present analysis were recruited from two nearby communities at one of the study sites, Baoshan district in Shanghai, China, during 2011 and 2013. All the eligible residents, men and women aged 40 years and above, were informed via phone notification or home visits by official residential committees, and invited to participate the filed physical examination at the primary health care center. A standardized questionnaire was adopted to collect information including lifestyle factors, disease and medical history; blood and urine sampling, anthropometric measurements, and 75-g oral glucose tolerances test (OGTT) were then performed.

Originally, 11,935 subjects (average age of 63.5 ± 13.5 years old and 4248 (35.6%) men) were recruited. We excluded individuals missing DNA sample (n = 143, 1.2%) or more than two single nucleotide polymorphism (SNP) genotypes (n = 319, 2.7%). Thus, 11473 (96.1%) participants were involved in the final analysis.

Written informed consent had been obtained from each participant. All methods were performed in accordance with the relevant guidelines and regulations. The Institutional Review Board of Rui-Jin Hospital, Shanghai Jiao Tong University School of Medicine, approved the study protocol.

### Anthropometric Information and Laboratory Measurements

General information including chronic diseases history, social demographic information, medications and lifestyle factors, such as tobacco and alcoholic consumption habits were collected with standardized questions. The current smoking or drinking status was defined as “yes” if the subject smoked cigarettes or consumed alcohol regularly in the past 6 months^22,24^. Body height and body weight were measured by trained researchers. BMI was calculated as body weight in kilograms divided by height squared in meters (kg/m^2^). Systolic and diastolic blood pressure (SBP and DBP) were measured by using an automated electronic device (OMRON Model HEM-752 FUZZY, Omron Company, Dalian, China) in triplicate on the same day after at least ten-min’s rest, and the average value of the three measurements was used for analysis.

Fasting and 2-hour postprandial plasma blood samples of all participants were obtained for biochemical detections; the former were collected after fasting at least for 10 hours, while the latter were collected at the 2-hour point after the OGTT test. Both fasting and 2-hour postprandial glucose (FPG and 2h-PG) were measured by using hexokinase method on a clinical chemistry diagnostic system (C16000, Abbott Laboratories, Otawara-shi, Japan). Serum concentrations of TG, TC, LDL-C and HDL-C were measured by using an auto-analyzer (ADVIA-1650 Chemistry System, Bayer Corporation, Germany). Serum fasting insulin was measured by using the immunoassay diagnostic system (I2000, Abbott Laboratories, Dallas, USA).

The detection of HbA1c levels was performed with the fasting plasma blood samples, which were collected by the Hemoglobin Capillary Collection System (Bio-Rad Laboratories). All samples were stored under the 2–8 centigrade environment and tested by the high-performance liquid chromatography using the VARIANT II Hemoglobin Testing System (Bio-Rad Laboratories) within 4 weeks after collection.

### Selection of Genetic Loci, Genotyping, and GRS Construction

17 common single nucleotide polymorphisms (SNPs) from 17 identified loci associated with HbA1c level were employed in the present study, which were all reported in recent genome wide association studies (GWASs)^25–28^, including: *HK1* rs7072268*, G6PC2* rs1402837*, SLC30A8* rs13266634*, TMPRSS6* rs855791, *FN3K* rs1046896, *ANK1* rs4737009, *G6PC2/ABCB11* rs552976, *MYO9B* rs11667918, *TMEM79* rs6684514, *HBS1L/MYB* rs9399137, *CYBA* rs9933309, *GCK* rs1799884, *ANK1* rs6474359, *SPTA1* rs2779116, *MTNR1B* rs1387153, *TCF7L2* rs7903146, and *CDKAL1* rs7772603.

All loci listed above reached a genome-wide significance level (*P* < 5 × 10^−8^); no linkage disequilibrium relationship was detected among one another; three SNPs failed to reach the Hardy-Weinberg equilibrium, they were *SLC30A8* rs13266634, *G6PC2/ABCB11* rs3755157, and *MYO9B* rs11667918 (*P* < 0.0001 for all the three). Most of the included SNPs in the present study were discovered or repeated in GWAS using East Asian population except for *HK1* rs7072268 and *G6PC2* rs1402837^27,29,30^. We also tested the association of all the selected SNPs with HbA1c in our samples and only 4 of 17, namely *HK1* rs7072268, *G6PC2/ABCB11* rs3755157, *ANK1* rs6474359, and *MTNR1B* rs1387153, were found insignificant. In addition, 8 loci were reported having significant associations with characters other than HbA1c level, including *SLC30A8* rs13266634, *TMPRSS6* rs855791, *TMEM79* rs6684514*, HBS1L/MYB* rs9399137, *GCK* rs1799884, *MTNR1B* rs1387153, *TCF7L2* rs7903146, and *CDKAL1* rs7772603. The pleiotropy information was initially searched on *GWAS Catalogue* (https://www.ebi.ac.uk/services), which is a GWAS database belongs to The European Molecular Biology Laboratory-The European Bioinformatic Institute (EMBL-EBI), and then verified in the referred literatures. The details were posted on the Supplemental Table 3.

To guarantee the power of the outcome, two sets of GRS, namely an expanded GRS and a conservative GRS, were built. The Expanded GRS was consisted of the whole 17 SNPs; while the Conservative GRS was consisted of 6 SNPs without reported pleiotropy, which all reached the HWE and had significant association with HbA1c either in our population or identified in other Eastern Asian populations before. All the GRSs were built with the additive genetic model^20^ by using a linear weighing of 0, 1 and 2 to genotypes containing 0, 1 or 2 risk alleles, respectively. Each allele was weighted by the effect size (β) of association with HbA1c summarized in the literatures^25–28^. Participants with more than two missing values of SNP (n = 319) were excluded and the ultimate population was finalized to 11,473; the mean score was utilized to represent scores of those with one or two missing values.

Blood white cells were collected for DNA extractions by using commercial blood genomic DNA extraction kit (OSR-M102-T1, TIANGEN BIOTECH CO, LTD, Beijing, China) on an automated nucleic acid extraction instrument (OSE-M48, TIANGEN BIOTECH CO, LTD, Beijing, China) according to manufacturer’s standard protocol. Specific assays were designed using the MassARRAY Assay Design software package (v3.1). Mass determination was carried out with the MALDI-TOF mass spectrometer and Mass ARRAY Type 4.0 software was used for data acquisition (SEQUENOM, CapitalBio Corporation, Beijing, China). Genotyping was performed in each subject. The minimum call rate was 99.3%. The concordance rate is more than 99% based on 100 duplicates genotyping.

### Statistical Analysis

The HOMA-β was calculated with the formula “20 × Fasting insulin (mIU/L)/[FPG (mmol/L)-3.5]”; HOMA-IR was calculated with the formula the “Fasting insulin (mIU/L) × FPG (mmol/L)/22.5”. Serum TG and HOMA-IR were logarithmically transformed for skewed distributions before statistical analysis. All the participants were divided into four groups based on HbA1c-GRS quartiles. Linear regression and Cochran-Armitage trend chi-square test were adopted to test the trend across quartiles for the continuous and categorical variables respectively. Multiple linear regression was adopted to test the associations of HbA1c-GRS with HbA1c and lipid profiles.

The HbA1c-GRS was selected as the IV and the IV estimator was adopted to measure the strength of the causal relationship between HbA1c and the four interested lipid indexes. The IV estimate of causal effect was calculated by the formula “β_MR_ = β_GRS-Outcome_/β_GRS-Exposure_”^31^.

We introduced five different methods in sensitivity analysis. First, two sets of unweighted GRS built with either 17 or 6 SNPs were adopted as IV. Second, causal estimates derived from individual SNPs were synthesized by inverse-variance weighted (IVW) linear regression model, which is commonly used in fixed-effect meta-analysis^32^. Third, weighted median method by combining data on multiple genetic variants into a single causal estimate which could maintain consistency even when up to 50% of the information comes from invalid instrumental variables^33^. Fourth, we performed the MR-Egger regression, basically ran the inverse-variance weighted model with the intercept unconstrained; the intercepts could be regarded as metrics of average pleiotropy effect across the chosen variants^34,35^. Lastly, the Mendelian randomization pleiotropy residual sum and outlier (MR-PRESSO) test were performed to a) detect horizontal pleiotropy (the MR-PRESSO global test), b) correct horizontal pleiotropy via outlier removal (the MR-PRESSO outlier test), and c) test the difference in the causal estimates before and after correction for outliers (the MR-PRESSO distortion test)^36^. To eliminate influence of anti-diabetic therapies and lifestyle changes after diagnosis of diabetes on HbA1c, the association and causal estimators of both the Expanded and Conservative HbA1c GRS with the four lipids were also tested in non-diabetic participants. Additionally, sensitivity analysis for GRSs by excluding SNPs with F-statistic < 10 in association with HbA1c were also performed to eliminate inaccuracy caused by possibly invalid instruments.

The statistical power of individual SNPs and the two GRSs in multiple regression analysis were calculated by G-power version 3.1.9.2 (https://www.softpedia.com/get/Science-CAD/G-Power.shtml). While the power of the two GRSs in MR analysis were calculated with an online power calculator (http://cnsgenomics.com/shiny/mRnd) particularly designed for MR analysis^37^. Association and MR analysis were performed on SAS version 9.4 (SAS Institute, Cary, NC) and R version 3.5.1 (The R Foundation for Statistical Computing). Sensitivity analysis with IVW, weighted median and MR-Egger methods were performed by R package “*Mendelianrandomization*”^38^, and the MR-PRESSO analysis by R package “*MRPRESSO*”^36^.

The cut-off of statistical significance was set at 0.05 for a two-sided *P* value.

## Results

### Characteristics of Study Participants

After the data trim, the analyzed participants included 4078 (35.54%) men and 7395 (64.46%) women with the average age of 63.2 ± 9.8 years old and the average BMI of 25.16 ± 3.37 kg/m^2^. The Expanded GRS was ranged from 8.40 to 29.10 with the mean of 18.35 ± 2.85; the Conservative GRS was ranged from 0.00 to 12.00 with the mean of 7.19 ± 1.73. For quartiles of both the Expanded GRS and the Conservative GRS, HbA1c-GRS was significantly associated with HbA1c. Besides, the increase of FPG, 2h-PBG, Log-TG, TC, LDL-C, HDL-C and the decrease of Log-HOMA-β were significantly associated with the increasing trend of Expanded-GRS quartiles (all *P* for trend < 0.05) (Table 1, Supplemental Table 1).

**Table 1:** Demographic, anthropometry and metabolic traits of the study participants categorized by quartiles of Expanded or Conservative GRS.

Characteristics	Quartile 1		Quartile 2		Quartile 3		Quartile 4		*P* for trend	
	E-GRS (n = 2,868)	C-GRS (n = 2,878)	E-GRS (n = 2,868)	C-GRS (n = 2,797)	E-GRS (n = 2,869)	C-GRS (n = 2,921)	E-GRS (n = 2,868)	C-GRS (n = 2,877)	E-GRS (n = 11,473)	C-GRS (n = 11,473)
HbA1c-GRS	14.76 ± 1.34	5.66 ± 0.89	17.48 ± 0.56	7.51 ± 0.36	19.31 ± 0.55	8.80 ± 0.40	21.98 ± 1.36	10.57 ± 0.88	<0.0001**	<0.0001**
HbA1c, mmol/mol (%)	40.93 ± 11.07 (5.89 ± 1.01)	41.31 ± 10.88 (5.94 ± 1.00)	42.02 ± 11.70 (5.99 ± 1.07)	42.04 ± 11.60 (6.00 ± 1.06)	42.24 ± 11.59 (6.01 ± 1.06)	42.39 ± 12.10 (6.02 ± 1.11)	43.34 ± 11.70 (6.12 ± 1.07)	43.02 ± 11.56 (6.06 ± 1.06)	<0.0001**	<0.0001**
Age, years	63.2 ± 9.9	63.3 ± 9.72	63.2 ± 9.6	63.1 ± 9.8	63.1 ± 9.8	63.1 ± 9.7	63.2 ± 9.7	63.2 ± 9.9	0.92	0.82
Male sex, n (%)	1,026 (35.8)	1,004 (34.9)	1,018 (35.5)	1,040 (36.4)	1,018 (35.5)	1,036 (35.5)	1,016 (35.4)	1018 (35.4)	0.79	0.63
BMI, kg/m2	25.2 ± 3.4	25.2 ± 3.4	25.2 ± 3.4	25.1 ± 3.4	25.1 ± 3.4	25.1 ± 3.4	25.1 ± 3.4	25.2 ± 3.3	0.39	0.77
SBP, mmHg	137 ± 20	137 ± 20	136 ± 20	137 ± 20	136 ± 21	137 ± 21	137 ± 20	137 ± 21	0.55	0.62
DBP, mmHg	77 ± 10	77 ± 10	77 ± 10	77 ± 10	77 ± 10	77 ± 10	77 ± 10	77 ± 10	0.24	0.68
FBG, mmol/L	5.93 ± 1.72	5.98 ± 1.69	6.02 ± 1.84	6.01 ± 1.79	6.00 ± 1.71	6.01 ± 1.77	6.06 ± 1.64	6.02 ± 1.67	0.04*	0.82
2-h PBG, mmol/L	8.72 ± 4.09	8.84 ± 4.07	8.85 ± 4.11	8.92 ± 4.14	8.93 ± 4.10	8.86 ± 4.05	9.04 ± 4.10	8.93 ± 4.14	0.02*	0.78
Log-HOMA-β	4.07 ± 0.60	4.04 ± 0.59	4.05 ± 0.60	4.04 ± 0.62	4.05 ± 0.59	4.02 ± 0.60	4.01 ± 0.59	4.02 ± 0.62	0.0003**	0.53
Log-HOMA-IR	0.47 ± 0.57	0.47 ± 0.59	0.48 ± 0.57	0.48 ± 0.59	0.49 ± 0.56	0.46 ± 0.58	0.50 ± 0.54	0.48 ± 0.59	0.28	0.45
Log-TG, mmol/L	0.27 ± 0.52	0.27 ± 0.52	0.27 ± 0.53	0.29 ± 0.54	0.30 ± 0.53	0.28 ± 0.51	0.30 ± 0.51	0.30 ± 0.52	0.02*	0.28
TC, mmol/L	4.92 ± 1.18	4.93 ± 1.17	4.91 ± 1.18	4.92 ± 1.20	4.94 ± 1.17	4.93 ± 1.18	4.99 ± 1.19	4.99 ± 1.17	0.02*	0.10
LDL-C, mmol/L	2.86 ± 0.88	2.87 ± 0.88	2.86 ± 0.88	2.85 ± 0.89	2.88 ± 0.88	2.88 ± 0.88	2.92 ± 0.90	2.92 ± 0.89	0.02*	0.03*
HDL-C, mmol/L	1.21 ± 0.33	1.22 ± 0.34	1.21 ± 0.33	1.21 ± 0.34	1.21 ± 0.33	1.20 ± 0.33	1.21 ± 0.33	1.21 ± 0.32	0.87	0.37

E-GRS, Expanded genetic risk score, was constructed with the whole 17 SNPs; C-GRS, Conservative genetic risk score, was constructed with 6 Conservative SNPs. SBP, systolic blood pressure; DBP, diastolic blood pressure; FBG, fasting blood glucose; PBG, post-prandial blood glucose; TG, triglyceride; TC, total cholesterol; LDL-C, low density lipoprotein cholesterol; HDL-C, high density lipoprotein cholesterol.

Continuous variables were displayed as mean ± SD; binary variables were displayed as n (%).The *P* for trend were derived from the linear regression for continuous variables or the trend test for binary variables between the interested traits and the quartiles of the corresponding GRSs. *P < 0.05; **P < 0.01.

### Association of HbA1c-GRS and HbA1c with Lipid Profiles

For the Expanded HbA1c-GRS, each SD (2.85 points) increased was associated with increment of 0.01-unit log-TG (β = 0.01, SE = 0.005, *P* = 0.005), 0.03 mmol/L TC (β = 0.03, SE = 0.01, *P* = 0.01), 0.02 mmol/L LDL-C (β = 0.02, SE = 0.008, *P* = 0.01) in the Model 1 which had age, sex and BMI adjusted. Whereas there was no significant association been detected (β = −0.001, SE = 0.003, *P* = 0.74) for HDL-C. After having systolic and diastolic blood pressure, smoking and drinking status, and physical activity status additionally adjusted (Model 2) and diabetes further adjusted as the full model (Model 3), no fundamental changes were observed. The R^2^ for the Expanded GRS in association analysis with HbA1c was 0.03.

In analysis using unit-SD (1.73 points) of the Conservative HbA1c-GRS, similar correlations with slightly attenuated magnitude of increment for log-TG (β = 0.005, SE = 0.003, *P* = 0.05), TC (β = 0.02, SE = 0.006, *P* = 0.01), and LDL-C (β = 0.01, SE = 0.005, *P* = 0.004) were observed; while the association with HDL-C (β = −0.005, SE = 0.002, *P* = 0.75) was still insignificant. The R^2^ for the Conservative GRS in association analysis with HbA1c was 0.01.

The observational associations between HbA1c level and lipid profiles were also tested. Under the fully adjusted model, each SD (1.06%) increment of HbA1c level was associated with 0.03 unit increase of log-TG (β = 0.03, SE = 0.005, *P* < 0.0001) and 0.03 mmol/L decrease of HDL-C (β = −0.03, SE = 0.004, *P* < 0.0001); while for TC (β = −0.02, SE = 0.01, *P* = 0.13) and LDL-C (β = 0.001, SE = 0.01, *P* = 0.89), no significant associations were observed **(**Supplemental Table 2).

### HbA1c-GRS and Lipid Profiles: The MR Analysis

**Figure 1:**
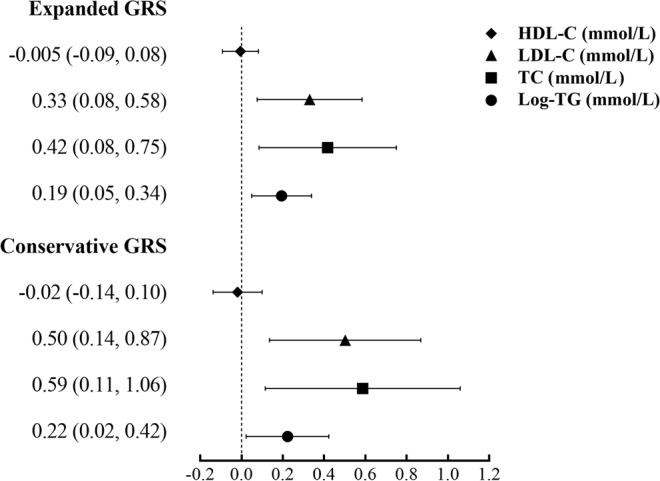
Forest plot for the causal association of HbA1c with lipids (mmol/L) by Expanded and Conservative GRSs. Statistics were presented as β coefficients and 95% confidential levels (CIs) of MR estimators; all data were calculated under the ultimate model with age, sex, BMI, systolic and diastolic blood pressure, smoking and drinking status, physical activity status, and diabetes adjusted.

The causal estimators of MR analysis for associations between HbA1c-GRS and lipid profiles were displayed on the Supplemental Table 4 and Fig. 1. Generally, the correlation tendency of the IV estimators agreed with that of associations between HbA1c-GRS and lipid profiles in all the three models. The genetically determined each 1-SD of HbA1c was related to 0.19 increment in log-TG (*P* = 0.009), 0.42 mmol/L TC (*P* = 0.01), and 0.33 mmol/L LDL-C (*P* = 0.01) with the Expanded HbA1c-GRS as the IV; and each 1-SD of HbA1c was related to 0.22 increase in log-TG (*P* = 0.03), 0.60 mmol/L TC (*P* = 0.01), and 0.51 mmol/L LDL-C (*P* = 0.007) with the Conservative HbA1c-GRS as the IV. Whereas no causal relationship was detected between HbA1c and HDL-C.

### Sensitivity Analysis

Sensitivity analysis were performed in the fully adjusted model with methods of un-weighted GRS, IVW, Weighted Median, MR-Egger, and MR-PRESSO for both Expanded and Conservative HbA1c-GRS, respectively. For TG, TC, and LDL-C, the beta-coefficients derived from the un-weighted GRS and IVW methods were close to the outcomes from the weighted HbA1c-GRS; and they all reached statistical significance (*P* < 0.05). According to the heterogeneity test reported by the “*Mendelianrandomization*” package^38^, the heterogeneity test statistic = 20.83 on 16 degrees of freedom, (*P* = 0.19), which indicated no significant heterogeneity. Therefore, the fixed-effects model was adopted. For outcomes of the Weighted Median method, LDL-C with the Expanded HbA1c-GRS and TC and LDL-C with the Conservative HbA1c-GRS were still significant. All Outcomes from MR-Egger method were failed to reach statistical significance, but the beta-coefficients for TG, TC, and LDL-C were approaching with those from the other methods; the intercepts of MR-Egger were all insignificant, which indicated no directional pleiotropy. For HDL-C, no significant causality was observed. The MR-PRESSO with Expanded HbA1c-GRS showed significant outcomes for Log-TG, TC, and LDL-C, and no significant horizontal pleiotropy for all 4 lipids. While with the Conservative HbA1c-GRS, the MR analysis for Log-TG was no longer significant and the global test showed significant outcome, which demonstrated detection of horizontal pleiotropy (Table 2). Further, the outlier test found a significant outlier (*rs1402837*, *P* = 0.04), but the distortion test did not find significant difference between the raw and outlier-corrected analysis (*P* = 0.52) (Supplemental Table 5).

**Table 2:** Sensitivity analysis for causal association of HbA1c with lipids (mmol/L) by unweighted GRS, IVW, Weighted median, MR-Egger and MR-PRESSO methods.

	Methods	Expanded GRS				Conservative GRS			
		β	SE	z- or t-statistic	*P* value	β	SE	z- or t-statistic	*P* value
Log-TG, (mmol/L)	**Weighted GRS**	0.19	0.07	2.62	0.009**	0.22	0.10	2.13	0.03*
	**Unweighted GRS**	0.22	0.07	2.98	0.003**	0.35	0.11	3.05	0.002**
	**IVW**	0.21	0.06	3.61	0.0003**	0.25	0.10	2.24	0.03*
	**Weighted Median**	0.13	0.11	1.18	0.23	0.22	0.14	1.57	0.12
	**Egger Estimate**	0.20	0.15	1.30	0.22	0.47	0.28	1.68	0.10
	**Egger Intercept**	0.0005	NA.	NA.	0.92	−0.0005	NA.	NA.	0.87
	**MR-PRESSO MR Analysis**	0.21	0.06	3.46	0.003**	0.24	0.11	2.18	0.07
	**Global Test**	NA.	NA.	NA.	0.25	NA.	NA.	NA.	0.043*.
TC, (mmol/L)	**Weighted GRS**	0.42	0.17	2.46	0.01*	0.60	0.24	2.44	0.01*
	**Unweighted GRS**	0.51	0.17	3.02	0.003**	0.67	0.25	2.66	0.008**
	**IVW**	0.48	0.15	3.17	0.002**	0.30	0.12	2.47	0.01*
	**Weighted Median**	0.38	0.22	1.73	0.08	0.30	0.13	2.40	0.02*
	**Egger Estimate**	0.31	0.37	0.81	0.42	0.32	0.27	1.19	0.24
	**Egger Intercept**	0.005	NA.	NA.	0.65	0.002	NA.	NA.	0.26
	**MR-PRESSO MR Analysis**	0.48	0.15	3.24	0.005**	0.30	0.12	2.53	0.04*
	**Global Test**	NA.	NA.	NA.	0.20	NA.	NA.	NA.	0.29
LDL-C, (mmol/L)	**Weighted GRS**	0.33	0.13	2.55	0.01**	0.51	0.19	2.70	0.007**
	**Unweighted GRS**	0.38	0.13	3.03	0.002**	0.53	0.19	2.74	0.006**
	**IVW**	0.38	0.10	3.62	0.0003**	0.25	0.10	2.41	0.02*
	**Weighted Median**	0.28	0.13	2.15	0.03*	0.24	0.10	2.45	0.02*
	**Egger Estimate**	0.33	0.25	1.32	0.21	0.33	0.28	1.57	0.10
	**Egger Intercept**	0.002	NA.	NA.	0.84	0.009	NA.	NA.	0.47
	**MR-PRESSO MR Analysis**	0.38	0.10	3.81	0.002**	0.25	0.10	2.53	0.04*
	**Global Test**	NA.	NA.	NA.	0.27	NA.	NA.	NA.	0.34
HDL-C, (mmol/L)	**Weighted GRS**	−0.005	0.04	0.10	0.92	−0.02	0.06	0.28	0.79
	**Unweighted GRS**	−0.02	0.04	0.57	0.58	−0.06	0.06	0.93	0.36
	**IVW**	−0.02	0.04	0.52	0.61	−0.009	0.04	0.23	0.82
	**Weighted Median**	0.05	0.09	0.56	0.58	−0.02	0.03	0.67	0.50
	**Egger Estimate**	−0.007	0.10	0.07	0.95	0.07	0.06	1.11	0.27
	**Egger Intercept**	−0.0005	NA.	NA.	0.87	−0.005	NA.	NA.	0.16
	**MR-PRESSO MR Analysis**	−0.02	0.04	−0.48	0.64	−0.009	0.04	−0.23	0.83
	**Global Test**	NA.	NA.	NA.	0.48	NA.	NA.	NA.	0.78

IVW, inverse-variance weighted method, Egger, MR-Egger method. MR-PRESSO, Mendelian randomization pleiotropy residual sum and outlier. For MR-PRESSO MR Analysis, outcomes of the outlier-corrected analysis were presented if outliers were detected, otherwise it would be the raw MR analysis. NA., not applicable.

The t statistics were calculated for MR-PRESSO while z statistics for the other analysis. *P* value were calculated from multivariable-adjusted linear regression. *P < 0.05; **P < 0.01. All data were calculated under the full model with age, sex, BMI, systolic and diastolic blood pressure, smoking and drinking status, physical activity status, and diabetes adjusted.

In non-diabetic participants (n = 8610), though the associations of the two GRSs with the four lipids were weakened, the results of causal association analyses were consistent with the analyses in the whole study sample, except for TC which was marginally significant in analysis with the Conservative GRS (*P* = 0.05) (Supplemental Table 6). In addition, according to the tests for association of individual SNPs with HbA1c, 7 SNPs had F-statistic < 10 **(**Supplemental Table 3**)**. While the sensitivity analysis by excluding them from both the Expanded and Conservative GRS (with 10 and 4 SNP remained respectively) showed no significant differences. The association and causal associations remained significant for Log-TG, TC, and LDL-C but not HDL-C after the 7 SNP been removed (Supplemental Table 7).

## Discussion

In the present MR analysis performed in a large sample size community-based Chinese population, we found that genetically determined HbA1c level was significantly associated with circulating TG, TC, and LDL-C, but not HDL-C, by using both Expanded and Conservative HbA1c-GRS as the IVs.

Although the associations between hyperlipidemia and hyperglycemia were extensively covered in observational studies^9–11^, the causal relationship for them is still indeterminate. A recent MR-analysis taking common 10 TG-associated SNPs as IV did not support the causal role of TG for increased fasting glucose^39^. Outcomes from another study using a hyperlipidemia risk score consisted of 95 lipid-associated SNPs displayed a negative association of the score with HbA1c level, which indicated protective effects of lipid-associated genes on hyperglycemia^40^. To our knowledge, this result is the first evidence for causality between HbA1c levels and circulating lipids profiles for East Asian populations. In a previous MR study, Ross *et al*. identified the causal effect of elevated HbA1c on the increased risk for CHD with 9 HbA1c-associated SNPs. Since TG and LDL-C have also been proven as the causal factors for increased CHD risk by prior MR analysis^6,41^, there existed an uncertainty about the interactions among hyperglycemia, hyperlipidemia, and CHD.

Recently, Xu *et al*. detected the causal association between cardiovascular risk factors including HbA1c level with risk of CHD. In which, it only identified a significant causal association of HbA1c increment with increased HDL-C level but not LDL-C, TG and TC for Caucasian populations^14^. However, in the present study, a strongly significant association of increased HbA1c level with TG was found in both the observational and the genetic association analysis. Moreover, distinct associations of the Expanded as well as the Conservative HbA1c-GRSs with serum TG were also detected in the MR analysis, which suggested the causal role of HbA1c increment for risk of higher circulating TG. In addition, remarkable associations of both the two GRSs with TC and LDL-C were also found in the current analysis. As for HDL-C, neither GRSs exhibited significant association, though previous observational studies showed inconsistence. Given the established one path of TG, TC and LDL-C with CHD, and the other of HbA1c with CHD, our results may imply that TG, TC, and LDL-C could be main mediators of the associations between HbA1c increment and increased risk of CHD for East Asians.

MR-analysis has been broadly adopted to probe exposure-outcome causality; whereas the robustness of the outcomes cannot be guaranteed until the three basic assumptions were met^42^. First, strong association of the exposure with the IVs must be identified before they were picked as proxies. Accordingly, all the 17 SNPs we used to create the GRSs were previously reported in genome wide association studies (GWASs) for exhibiting significant correlations with HbA1c levels. Secondly, the IVs should be independent from potential confounders. In the present study, several SNPs selected were reported for associations with traits other than HbA1c. Of these traits, some were widely known for associations with lipids, like type 2 diabetes, BMI, and metabolic syndrome, while for the others like iron status, mean corpuscular hemoglobin and mean corpuscular volume, their correlations with lipids were yet clarified and not frequently discussed. However, to minimize the possibility of pleiotropy and keep robustness, we still tended to rule out all SNP with associated traits other than HbA1c to construct the Conservative GRS. This strategy might loss some genetic effect in causal estimation, but could reduce the possibility to violate the basic assumption of MR analysis in the greatest extent. Compared with the results get from the Expanded GRSs, the data derived from the Conservative one exhibited slight but unobtrusive fluctuations. Predictably, the 95% CI of the Conservative outcomes were significantly widened for its purpose of ensuring robustness. But overall, there were no fundamental divergence spotted in the comparison. Lastly, the IV should neither correlate with the interested outcome through any other paths than the exposure. However, to have this assumption verified would be impractical. It is impossible to rule out the possibility that the selected variants are affecting the interested outcomes via the mechanisms besides the exposure. Consequently, we adopted multivariable analysis to adjust the effects of some common risk factors for dyslipidemia, including adiposity, blood pressure, smoking and drinking status, physical activity, and diabetes. Slight variations displayed as the adjusted factors added from the basal model to the full model, but no remarkable changes were observed. Therefore, we hypothesize that the association between genetically determined HbA1c levels with increased circulating TG, TC, and LDL-C is due to a direct consequence of increased HbA1c level.

A previous experimental study demonstrated that hyperglycemia status could suppress fatty acid oxidation and accelerate TG accumulation^43^. This was confirmed by another recent experiment on trophoblast cell line, which identified significant changes on multiple lipid metabolic pathways, including fatty acid β-oxidation, phospholipid metabolism and phosphatidylinositol phosphate signaling, in response to high glucose environment^44^. These findings may suggest an interference effect of hyperglycemia on lipidic metabolism. Lately, Laugier-Robiolle *et al*. investigated the association between proprotein convertase subtilisin/kexin type 9 (PCSK9), a post-transcriptional inhibitor of LDL-receptor, and plasma LDL-C in T1D patients classified by HbA1c; a better glycemic control was found able to abolish the positive relationship between PCSK9 and LDL-C in T1D patients^45^. This was in accordance with another recent study on a cohort of T1D children, in which the trajectories of LDL, and non-HDL cholesterol levels went up modestly as HbA1c increased across their ages from childhood into young adulthood ranged from 1.0 to 19.5 years with a median of 9.3. Of late years, increasing studies regarding with the association of HbA1c and dyslipidemia in Eastern Asian populations emerged. A longitudinal study followed for 15 years in a Japanese cohort identified significant associations of HbA1c with higher TC and lower HDL-C^46^. Recently, a clinical trial for DPP-4 inhibitor gemigliptin in a Korean population showed that TC and LDL-C but not TG and HDL-C in the gemigliptin group were reduced significantly compared with the placebo group^12^. However, the detailed mechanism for the hyperlipidemia-promoting effect of HbA1c is still incompletely understood.

The strength of the current study was evidenced by a well-defined community setting, fair sized sample volume, desirable population homogeneity, and multiple sensitivity analysis. Meanwhile, we acknowledge the following limitations in our study. Firstly, we did not consider the impact caused by anti-dyslipidemia treatment and therefore might underestimate the causal effect. However, in accordance with the experience from our previous studies^17,22,23,47^ and other epidemiological investigations^18^, the proportion of anti-dyslipidemia treatment is small, for which its influence could probably be omitted. Secondly, the variants we used in GRS construction were all common variants; the heritability of low frequency and rare variants could hardly be evaluated. Thirdly, despite that we introduced the Conservative HbA1c-GRS to preclude the known pleiotropy, interference from the residual confounding were still beyond control. Fourthly, measuring inaccuracy might happen because of ignored biochemical changes of lipids after been generated in blood, for instance, the circulating LDL-C levels might be underrated because of increased oxidization rate in hyperglycemia environment^48,49^. Fifthly, the present analysis was performed in the middle aged and elderly participants. We adopted the sample in this age group to gain a higher study power given our emphasis on chronic metabolic disease, such as type 2 diabetes, hypertension and dyslipidemia, etc. Although we adjusted age in all models, it should be cautious to generalize the present results to younger population. And finally, in view of the highly consistent composition of the population analyzed, it should be more careful to apply our findings to interpret cases of other ethnicities or ethnic groups.

In conclusion, our study supported a causal effect of the increased HbA1c for increments of TG, TC, and LDL-C levels in a Chinese population. However, we cannot eliminate all the potential pleiotropy even with the use of Conservative GRS. Thus, it should be cautiously applied to further researches or clinical decisions. Meantime, experimental studies to elucidate the biological and biochemical mechanisms are anticipated.

## Supplementary information

online supplementary materials

## Data Availability

The dataset generated during and/or analyzed during the current study are not publicly available due to some other unpublished studies based on this dataset, but are available from the corresponding author on reasonable request.
