# Supplementary material for: Mendelian Randomization Analysis Support Causal Associations of HbA1c with Circulating Triglyceride, Total and Low-density Lipoprotein Cholesterol in a Chinese Population
Source: Sci Rep. 2019 Apr 2;9:5525. doi: 10.1038/s41598-019-41076-6 (PMC6445078; doi:10.1038/s41598-019-41076-6)
Supplement: Supplementary file 1 — online supplementary materials [file 41598_2019_41076_MOESM1_ESM.docx]

**Online Supplemental Materials**

**Mendelian Randomization Analysis Support Causal Associations of HbA1c with Circulating Triglyceride, Total and Low-density Lipoprotein Cholesterol in a Chinese Population**

Xu Jia^1,2†^, Min Xu^1,2†^, Zhiyun Zhao^1,2†^, Liping Xuan^1,2^, Yanan Hou^1,2^, Tiange Wang^1,2^, Mian Li^1,2^, Yu Xu^1,2^, Jieli Lu^1,2^, Yufang Bi^1,2^, Weiqing Wang^1,2^, Yuhong Chen^1,2^

^†^These authors contributed equally to this study.

| **Characteristics** | **Mean** | **Standard Deviation** | **Median** | | **Maximum** | **Minimum** |
| --- | --- | --- | --- | --- | --- | --- |
| **Expanded GRS** | 18.35 | 2.85 | 18.36 | | 29.10 | 8.40 |
| **Conservative GRS** | 7.19 | 1.73 | 7.04 | | 12.00 | 0.00 |
| **HbA1c, mmol/mol (%)** | 42.13 (6.00) | 11.55 (1.06) | 38.80 (5.70) | | 110.93 (12.3) | 9.29 (3.00) |
| **Age, years** | 63.17 | 9.75 | 62.00 | | 93.00 | 40.00 |
| **BMI, kg/m2** | 25.16 | 3.37 | 25.02 | | 35.99 | 11.89 |
| **SBP, mmHg** | 136.85 | 20.31 | 136.00 | | 250.00 | 72.00 |
| **DBP, mmHg** | 77.19 | 10.24 | 77.00 | | 159.00 | 41.00 |
| **FBG, mmol/L** | 6.00 | 1.73 | 5.52 | | 8.30 | 3.17 |
| **2h PBG, mmol/L** | 8.89 | 4.10 | 7.65 | | 18.61 | 8.06 |
| **Log-HOMA-IR** | 0.49 | 0.56 | 0.47 | | 1.17 | 0.00 |
| **Log-HOMA-β** | 4.04 | 0.59 | 4.09 | | 5.63 | 0.86 |
| **Log-TG, mmol/L** | 0.28 | 0.52 | 0.25 | | 0.76 | 0.07 |
| **TC, mmol/L** | 4.94 | 1.18 | 4.95 | | 12.51 | 0.50 |
| **LDL-C, mmol/L** | 2.88 | 0.89 | 2.85 | | 8.33 | 0.24 |
| **HDL-C, mmol/L** | 1.21 | 0.33 | 1.18 | | 3.04 | 0.14 |
|  | **N** | | | **Percentage (%)** | | |
| **Male sex, n (%)** | 4078 | | | 35.54 | | |
| **Diabetes** | 2863 | | | 24.95 | | |
| **Current Smoking, n (%)** | 1661 | | | 14.1 | | |
| **Current Drinking, n (%)** | 1052 | | | 8.93 | | |
| **Modest PA, n (%)** | 1994 | | | 16.93 | | |
| **Vigorous PA, n (%)** | 696 | | | 5.91 | | |

**Supplemental Table 1.** Demographic, anthropometry and metabolic traits of the study population

The expanded GRS were consisted of 17 SNPs, and the conservative GRS were consisted of 6 SNPs.

|  | **Model 1** | | | | | **Model 2** | | | | | **Model 3** | | | | |
| --- | --- | --- | --- | --- | --- | --- | --- | --- | --- | --- | --- | --- | --- | --- | --- |
| **Traits** | **β** | **SE** | **t-statistic** | ***P* value** | **Power** | **β** | **SE** | **t-statistic** | ***P* value** | **Power** | **β** | **SE** | **t-statistic** | ***P* value** | **Power** |
| **Associations of** **Expanded GRS with HbA1c and lipids** | | | | | | | | | | | | | | | |
| **HbA1c, %** | 0.08 | 0.01 | 8.27 | <0.0001 ** | 100% | 0.08 | 0.01 | 8.38 | <0.0001 ** | 100% | 0.07 | 0.008 | 8.13 | <0.0001** | 100% |
| **Log-TG, mmol/L** | 0.01 | 0.005 | 2.79 | 0.005 ** | 100% | 0.01 | 0.005 | 3.01 | 0.003 ** | 100% | 0.01 | 0.005 | 2.76 | 0.006** | 100% |
| **TC, mmol/L** | 0.03 | 0.01 | 2.47 | 0.01* | 100% | 0.03 | 0.01 | 2.59 | 0.01* | 100% | 0.03 | 0.01 | 2.57 | 0.01* | 100% |
| **LDL-C, mmol/L** | 0.02 | 0.008 | 2.58 | 0.01* | 100% | 0.02 | 0.008 | 2.68 | 0.008 ** | 100% | 0.02 | 0.008 | 2.68 | 0.008** | 100% |
| **HDL-C, mmol/L** | -0.001 | 0.003 | -0.33 | 0.74 | 100% | -0.0007 | 0.003 | -0.25 | 0.81 | 100% | -0.0003 | 0.003 | -0.10 | 0.92 | 100% |
| **Associations of Conservative GRS with HbA1c and lipids** | | | | | | | | | | | | | | | |
| **HbA1c, %** | 0.03 | 0.006 | 4.86 | <0.0001 ** | 100% | 0.03 | 0.006 | 4.90 | <0.0001 ** | 100% | 0.03 | 0.005 | 6.08 | <0.0001 ** | 100% |
| **Log-TG, mmol/L** | 0.005 | 0.003 | 1.97 | 0.05* | 100% | 0.006 | 0.003 | 2.25 | 0.02 * | 100% | 0.006 | 0.003 | 2.27 | 0.02* | 100% |
| **TC, mmol/L** | 0.02 | 0.006 | 2.46 | 0.01* | 100% | 0.02 | 0.006 | 2.66 | 0.008** | 100% | 0.02 | 0.006 | 2.66 | 0.008** | 100% |
| **LDL-C, mmol/L** | 0.01 | 0.005 | 2.87 | 0.004** | 100% | 0.01 | 0.005 | 3.01 | 0.003* | 100% | 0.01 | 0.005 | 3.01 | 0.003** | 100% |
| **HDL-C, mmol/L** | -0.0005 | 0.002 | -0.32 | 0.75 | 100% | -0.0005 | 0.002 | -0.27 | 0.78 | 100% | -0.0005 | 0.002 | -0.28 | 0.78 | 100% |
| **Associations of HbA1c with lipids** | | | | | | | | | | | | | | | |
| **Log-TG, mmol/L** | 0.05 | 0.004 | 11.82 | <0.0001 ** | 100% | 0.06 | 0.005 | 11.78 | <0.0001 ** | 100% | 0.03 | 0.005 | 5.57 | <0.0001** | 100% |
| **TC, mmol/L** | -0.008 | 0.01 | -0.77 | 0.44 | 100% | -0.007 | 0.01 | -0.66 | 0.51 | 100% | -0.02 | 0.01 | -1.53 | 0.13 | 100% |
| **LDL-C, mmol/L** | -0.004 | 0.008 | -0.53 | 0.59 | 100% | -0.005 | 0.008 | -0.58 | 0.56 | 100% | 0.001 | 0.01 | 0.14 | 0.89 | 100% |
| **HDL-C, mmol/L** | -0.03 | 0.003 | -11.77 | <0.0001 ** | 100% | -0.03 | 0.003 | -11.28 | <0.0001 ** | 100% | -0.03 | 0.003 | -9.01 | <0.0001** | 100% |

**Supplemental Table 2.** Associations of HbA1c (%) and GRSs with HbA1c and lipids (mmol/L)

*P* value were calculated from multivariable-adjusted linear regression models. *: *P* < 0.05; **: *P* < 0.01.

Model 1 adjusted for age, sex, and BMI; Model 2 further adjusted for systolic and diastolic blood pressure, smoking and drinking status, and physical activity status based on Model 1; Model 3 further adjusted for diabetes.

| **Locus** | **SNP** | **Chr.** | **Position** | ***P* for**  **HWE** | **Risk allele/other** | **EAF** | **Reported pleiotropy** | **Verification in the EA population** | **β_0_** | **β** | **SE** | **t-statistic** | **F-statistic** | ***P* value** | **R^2^** | **Power** |
| --- | --- | --- | --- | --- | --- | --- | --- | --- | --- | --- | --- | --- | --- | --- | --- | --- |
| ***HK1*** | rs7072268 | 10 | 69340157 | 0.82 | G/A | 0.07 | No | No | 0.02 | 0.007 | 0.007 | 0.98 | 0.97 | 0.33 | 0.0001 | 20.52% |
| ***G6PC2*** | rs1402837 | 2 | 168900844 | 0.24 | G/A | 0.16 | No | No | 0.02 | 0.02 | 0.006 | 2.67 | 7.13 | 0.008 | 0.0008 | 86.93% |
| ***SLC30A8*** | rs13266634 | 8 | 117172544 | <0.0001 | G/A | 0.33 | T2D | Yes | 0.02 | 0.01 | 0.006 | 1.98 | 3.92 | 0.046 | 0.0005 | 62.74% |
| ***TMPRSS6*** | rs855791 | 22 | 37066896 | 0.32 | A/G | 0.27 | Iron status  /MCH | Yes | 0.03 | 0.03 | 0.006 | 4.82 | 23.21 | <0.0001 | 0.003 | 99.98% |
| ***FN3K*** | rs1046875 | 17 | 82727550 | 0.31 | A/G | 0.23 | No | Yes | 0.08 | 0.04 | 0.006 | 6.02 | 36.32 | <0.0001 | 0.004 | 99.99% |
| ***ANK1*** | rs4737009 | 8 | 41772887 | 0.19 | A/G | 0.27 | No | Yes | 0.08 | 0.03 | 0.006 | 4.17 | 17.37 | <0.0001 | 0.002 | 99.78% |
| ***G6PC2/ABCB11*** | rs3755157 | 2 | 168935661 | <0.0001 | T/C | 0.97 | No | Yes | 0.07 | 0.04 | 0.02 | 1.53 | 2.33 | 0.13 | 0.0003 | 42.22% |
| **MYO9B** | rs11667918 | 19 | 17121689 | <0.0001 | C/T | 0.37 | No | Yes | 0.06 | 0.03 | 0.006 | 5.76 | 33.14 | <0.0001 | 0.004 | 99.99% |
| **TMEM79** | rs6684514 | 1 | 156285665 | 0.62 | G/A | 0.56 | MCH | Yes | 0.09 | 0.04 | 0.007 | 6.14 | 37.66 | <0.0001 | 0.004 | 99.99% |
| **HBS1L/MYB** | rs9399137 | 6 | 135097880 | 0.44 | T/C | 0.50 | Platelet  /MCV/MCH | Yes | 0.07 | 0.03 | 0.007 | 4.04 | 16.37 | <0.0001 | 0.002 | 99.66% |
| **CYBA** | rs9933309 | 16 | 88778524 | 0.34 | C/T | 0.42 | No | Yes | 0.07 | 0.02 | 0.007 | 3.67 | 13.44 | 0.0002 | 0.002 | 98.84% |
| ***GCK*** | rs1799884 | 7 | 44189469 | 0.73 | T/C | 0.05 | Glycemic traits/Metabolite | Yes | 0.12 | 0.04 | 0.008 | 5.35 | 28.62 | <0.0001 | 0.003 | 99.99% |
| ***ANK1*** | rs6474359 | 8 | 41691676 | 0.06 | T/C | 0.92 | No | Yes | 0.06 | -0.004 | 0.02 | 0.13 | 0.08 | 0.78 | 0.000002 | 5.26% |
| ***SPTA1*** | rs2779116 | 1 | 158615625 | 0.31 | T/C | 0.19 | No | Yes | 0.02 | 0.03 | 0.006 | 5.13 | 26.32 | <0.0001 | 0.003 | 99.99% |
| ***MTNR1B*** | rs1387153 | 11 | 92940662 | 0.19 | T/C | 0.17 | T2D/FBG/MS | Yes | 0.03 | 0.01 | 0.006 | 1.75 | 3.06 | 0.08 | 0.0004 | 52.44% |
| ***TCF7L2*** | rs7903146 | 10 | 112998590 | 0.13 | T/C | 0.01 | T2D/FBG  /MS/BMI | Yes | 0.05 | 0.04 | 0.02 | 2.32 | 5.37 | 0.02 | 0.0006 | 76.31% |
| ***CKDAL1*** | rs7772603 | 6 | 20685255 | 0.75 | T/C | 0.16 | BMI/FBG | Yes | 0.06 | 0.02 | 0.006 | 3.78 | 14.26 | 0.0002 | 0.002 | 99.18% |

**Supplemental Table 3.** SNP Information, pleiotropy and association with HbA1c (%)

SNP, single nucleotide polymorphism; GRS, genetic risk statistic; Chr., chromosome; HWE, Hardy-Weinberg equilibrium. T2D, type 2 diabetes; MCH, mean corpuscular hemoglobin; MCV, mean corpuscular volume; FBG, fasting blood glucose; MS, metabolic syndrome; BMI, body mass index; EA, East Asian population.

The SNPs were shown in risk allele and effect allele frequency (EAF). Information of reported pleiotropy was retrieved on the *GWAS Catalogue (https://www.ebi.ac.uk/services).* The verification in EA population means the SNP was either discovered or replicated in GWAS based on East Asian population. The β_0_ were derived from prior GWAS and used as the weight in GRS construction. The β, SE, t statistic, F statistic, and *P* value were calculated using the non-diabetic individuals in population of the present study (n=8610); the t statistic was derived from multivariable-adjusted linear regression models, while the F statistic were calculated in corresponding variance analysis. *P* value and R^2^ were calculated from linear regression models.

**Supplemental Table 4.** Causal association of HbA1c with lipids (mmol/L) by the Expanded and Conservative GRS

| **Characters** | **Model 1** | | | | | **Model 2** | | | | | **Model 3** | | | | |
| --- | --- | --- | --- | --- | --- | --- | --- | --- | --- | --- | --- | --- | --- | --- | --- |
|  | **β** | **SE** | **z-statistic** | ***P* value** | **Power** | **β** | **SE** | **z-statistic** | ***P* value** | **Power** | **β** | **SE** | **z-statistic** | ***P* value** | **Power** |
| **Causal associations of Expanded GRS with lipids** | | | | | | | | | | | | | | | |
| **Log-TG, mmol/L** | 0.16 | 0.06 | 2.65 | 0.008** | 98% | 0.18 | 0.06 | 2.82 | 0.005** | 99% | 0.19 | 0.07 | 2.62 | 0.009** | 100% |
| **TC, mmol/L** | 0.33 | 0.14 | 2.36 | 0.02* | 100% | 0.35 | 0.14 | 2.47 | 0.01* | 100% | 0.42 | 0.17 | 2.46 | 0.01* | 100% |
| **LDL-C, mmol/L** | 0.26 | 0.11 | 2.47 | 0.01* | 100% | 0.27 | 0.11 | 2.52 | 0.01* | 100% | 0.33 | 0.13 | 2.55 | 0.01* | 100% |
| **HDL-C, mmol/L** | -0.01 | 0.04 | 0.33 | 0.76 | 87% | -0.009 | 0.04 | 0.25 | 0.82 | 6% | -0.005 | 0.04 | 0.10 | 0.92 | 5% |
| **Causal associations of Conservative GRS with lipids** | | | | | | | | | | | | | | | |
| **Log-TG, mmol/L** | 0.20 | 0.11 | 1.83 | 0.07 | 84% | 0.22 | 0.11 | 2.04 | 0.04* | 90% | 0.22 | 0.10 | 2.13 | 0.03* | 89% |
| **TC, mmol/L** | 0.56 | 0.26 | 2.20 | 0.03* | 100% | 0.60 | 0.26 | 2.34 | 0.02* | 100% | 0.60 | 0.24 | 2.44 | 0.01* | 100% |
| **LDL-C, mmol/L** | 0.50 | 0.20 | 2.47 | 0.01* | 100% | 0.51 | 0.20 | 2.56 | 0.01* | 100% | 0.51 | 0.19 | 2.70 | 0.007** | 100% |
| **HDL-C, mmol/L** | -0.02 | 0.06 | 0.32 | 0.76 | 7% | -0.02 | 0.06 | 0.27 | 0.80 | 7% | -0.02 | 0.06 | 0.28 | 0.79 | 7% |

The MR estimates β were calculated by the formula “β_MR_ = β_GRS-Outcome_/β_GRS-Exposure_”; the SE were calculated by the formula “SE_MR_ = SE_GRS-Outcome_/β_GRS-Exposure_”, and *P* value were calculated from multivariable-adjusted linear regression. *: *P* < 0.05; **: *P* < 0.01. Power were calculated with R^2^ for association of GRS with HbA1c.

Model 1 adjusted for age, sex, and BMI; Model 2 further adjusted for systolic and diastolic blood pressure, smoking and drinking status, and physical activity status based on Model 1; Model 3 further adjusted for diabetes.

| **Test** | **Raw Analysis** | | | | **Global Test** | | **Outlier Test** | | **Distortion Test** | **Outlier-corrected Analysis** | | | |
| --- | --- | --- | --- | --- | --- | --- | --- | --- | --- | --- | --- | --- | --- |
|  | **β** | **SE** | **t-statistic** | ***P* value** | **RSS-obs** | ***P* value** | **Outlier** | ***P* value** | ***P* value** | **β** | **SE** | **t-statistic** | ***P* value** |
| **MR-PRESSO analysis with 17 SNPs** | | | | | | | | | | | | | |
| **Log-TG, mmol/L** | 0.21 | 0.06 | 3.46 | 0.003** | 22.50 | 0.25 | No outlier detected | | NA. | No outlier detected | | | |
| **TC, mmol/L** | 0.48 | 0.15 | 3.24 | 0.005** | 23.96 | 0.20 | No outlier detected | | NA. | No outlier detected | | | |
| **LDL-C, mmol/L** | 0.38 | 0.10 | 3.81 | 0.002** | 21.96 | 0.27 | No outlier detected | | NA. | No outlier detected | | | |
| **HDL-C, mmol/L** | -0.02 | 0.04 | -0.48 | 0.64 | 18.05 | 0.48 | No outlier detected | | NA. | No outlier detected | | | |
| **MR-PRESSO analysis with 6 SNPs** | | | | | | | | | | | | | |
| **Log-TG, mmol/L** | 0.25 | 0.10 | 2.57 | 0.04* | 18.88 | 0.043* | rs1402837 | 0.04* | 0.52 | 0.24 | 0.11 | 2.18 | 0.07 |
| **TC, mmol/L** | 0.30 | 0.12 | 2.53 | 0.04* | 10.00 | 0.29 | No outlier detected | | NA. | No outlier detected | | | |
| **LDL-C, mmol/L** | 0.25 | 0.10 | 2.53 | 0.04* | 9.93 | 0.34 | No outlier detected | | NA. | No outlier detected | | | |
| **HDL-C, mmol/L** | -0.009 | 0.04 | -0.23 | 0.83 | 4.63 | 0.78 | No outlier detected | | NA. | No outlier detected | | | |

**Supplemental Table 5**. Sensitivity analysis for causal association of HbA1c with lipids (mmol/L) by MR-PRESSO method

MR-PRESSO, Mendelian randomization pleiotropy residual sum and outlier; RSS-obs, observed residual sum of squares; NA., not applicable. The outlier-corrected analysis would be performed if outliers were detected, and distortion test would be performed if the detected outliers were significant.

**Supplemental Table 6.** Sensitivity analysis for causal association of HbA1c with lipids (mmol/L) in non-diabetic population

|  | **Expanded GRS** | | | | **Conservative GRS** | | | |
| --- | --- | --- | --- | --- | --- | --- | --- | --- |
|  | **β** | **SE** | **t-statistic** | ***P* value** | **β** | **SE** | **t-statistic** | ***P* value** |
| **Associations of GRS with HbA1c and lipids** | | | | | | | | |
| **HbA1c, %** | 0.07 | 0.004 | 16.04 | <0.0001** | 0.02 | 0.002 | 9.41 | <0.0001** |
| **Log-TG, mmol/L** | 0.01 | 0.005 | 2.53 | 0.01* | 0.006 | 0.003 | 2.07 | 0.04* |
| **TC, mmol/L** | 0.02 | 0.01 | 1.25 | 0.21 | 0.01 | 0.005 | 2.00 | 0.05* |
| **LDL-C, mmol/L** | 0.01 | 0.009 | 1.41 | 0.16 | 0.01 | 0.005 | 2.05 | 0.04* |
| **HDL-C, mmol/L** | -0.001 | 0.003 | -0.36 | 0.72 | -0.0009 | 0.002 | -0.47 | 0.63 |
| **Causal Associations of GRS with lipids** | | | | | | | | |
| **Log-TG, mmol/L** | 0.19 | 0.07 | 2.62 | 0.009* | 0.27 | 0.13 | 2.02 | 0.04* |
| **TC, mmol/L** | 0.42 | 0.17 | 2.46 | 0.01* | 0.48 | 0.25 | 1.95 | 0.05 |
| **LDL-C, mmol/L** | 0.33 | 0.13 | 2.55 | 0.01* | 0.47 | 0.24 | 2.01 | 0.04* |
| **HDL-C, mmol/L** | -0.005 | 0.04 | -0.10 | 0.92* | -0.04 | 0.09 | -0.48 | 0.64 |

Data are β, SE. The *P* values were adjusted age, sex, BMI, SBP, DBP, smoking and drinking status, and physical activity.

**Supplemental Table 7.** Sensitivity analysis for causal association of HbA1c (%) with lipids (mmol/L) by GRSs excluding SNPs with F-statistic < 10

|  | **10 SNP consisted GRS** | | | | **4 SNP consisted GRS** | | | |
| --- | --- | --- | --- | --- | --- | --- | --- | --- |
|  | **β** | **SE** | **t-statistic** | ***P* value** | **β** | **SE** | **t-statistic** | ***P* value** |
| **Associations of GRS with HbA1c and lipids** | | | | | | | | |
| **HbA1c, %** | 0.03 | 0.00 | 8.10 | <0.0001** | 0.03 | 0.01 | 6.10 | <.0001** |
| **Log-TG, mmol/L** | 0.02 | 0.01 | 2.64 | 0.01* | 0.02 | 0.01 | 2.64 | 0.01* |
| **TC, mmol/L** | 0.03 | 0.01 | 2.25 | 0.02* | 0.03 | 0.01 | 2.25 | 0.02* |
| **LDL-C, mmol/L** | 0.02 | 0.01 | 2.48 | 0.01* | 0.02 | 0.01 | 2.48 | 0.01* |
| **HDL-C, mmol/L** | 0.00 | 0.00 | -0.17 | 0.86 | 0.00 | 0.00 | -0.17 | 0.86 |
| **Causal Associations of GRS with lipids** | | | | | | | | |
| **Log-TG, mmol/L** | 0.53 | 0.21 | 2.51 | 0.01* | 0.47 | 0.19 | 2.42 | 0.02* |
| **TC, mmol/L** | 1.05 | 0.48 | 2.17 | 0.03* | 0.92 | 0.44 | 2.11 | 0.03* |
| **LDL-C, mmol/L** | 0.88 | 0.37 | 2.37 | 0.02* | 0.77 | 0.34 | 2.30 | 0.02* |
| **HDL-C, mmol/L** | -0.02 | 0.13 | -0.17 | 0.87 | -0.02 | 0.11 | -0.17 | 0.87 |

Data are β, SE. The P values were adjusted age, sex, BMI, SBP, DBP, smoking and drinking status, and physical activity.

**Supplemental Figure 1**. Fitting charts for sensitivity analysis for causal association of HbA1c with lipids (mmol/L) by IVW, MR-Egger, and weighted median methods


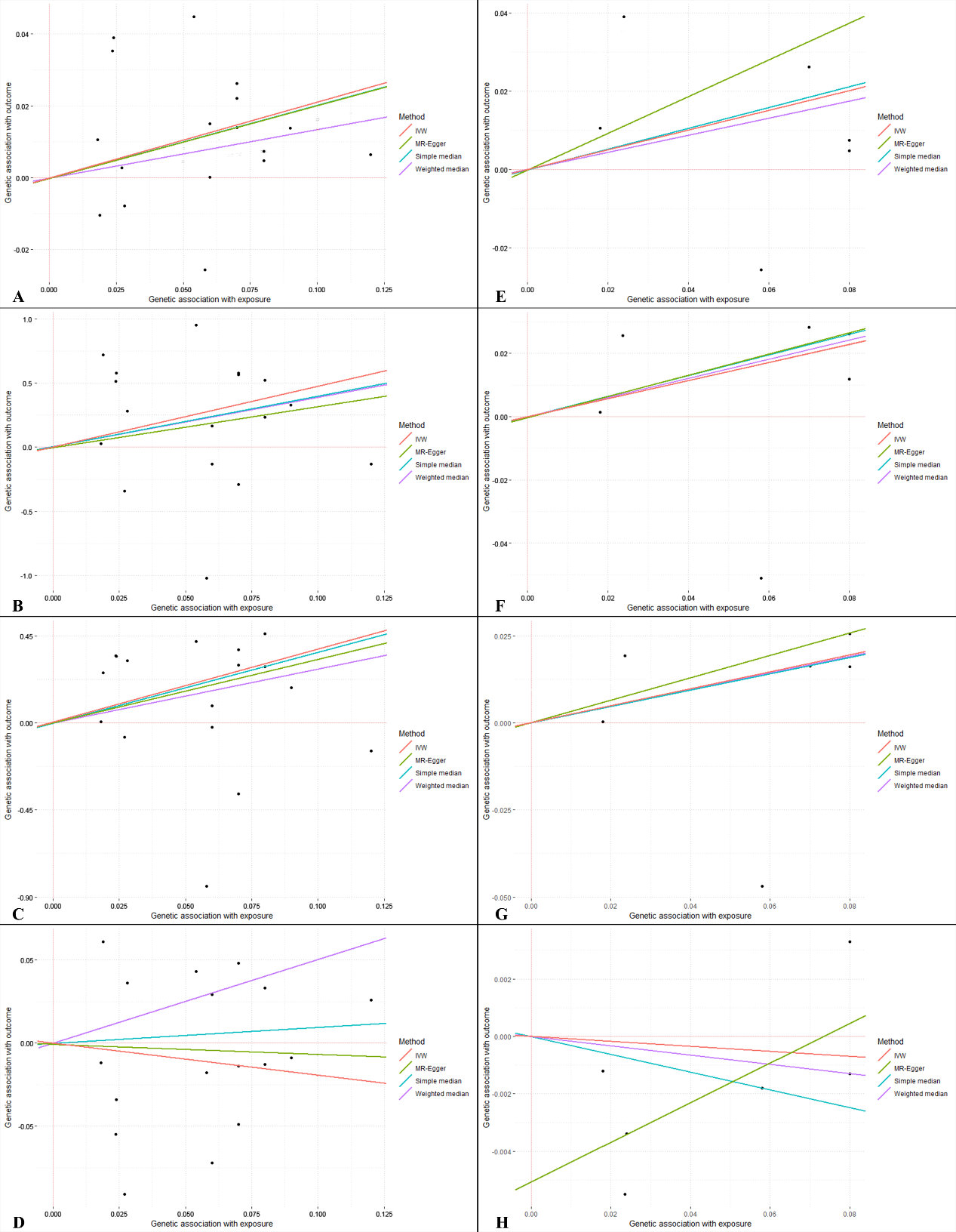


The fitting charts were generated by R with the package of “Mendelian Randomization”. All methods were performed with the Model 3.

Panel A-D, analysis for log-TG, TC, LDL, and HDL respectively using 17 SNP; Panel E-H, analysis for log-TG, TC, LDL, and HDL respectively 6 SNP.
